# Identification of SLC26A4 c.919-2A>G compound heterozygosity in hearing-impaired patients to improve genetic counseling

**DOI:** 10.1186/1479-5876-10-225

**Published:** 2012-11-14

**Authors:** Qi Li, Qing-wen Zhu, Yong-yi Yuan, Sha-sha Huang, Dong-yi Han, De-liang Huang, Pu Dai

**Affiliations:** 1 Division of Pediatric Otolaryngology, Nanjing Children’s Hospital, Nanjing Medical University, Nanjing, Jiangsu, 210008, China; 2 Department of Otolaryngology - Head and Neck Surgery, PLA General Hospital, Beijing, 100853, China; 3 Department of Otolaryngology - Head and Neck Surgery, Hainan Branch of PLA General Hospital, Sanya, 572000, China

## Abstract

**Background:**

Mutations in the SLC26A4 gene, which encodes the anion transporter, pendrin, are a major cause of autosomal recessive non-syndromic hearing loss (NSHL) in some Asian populations. SLC26A4 c.919-2A>G (IVS7-2A>G) is the most common mutation in East Asian deaf populations. To provide a basis for improving the clinical diagnosis of deaf patients, we evaluated 80 patients with the SLC26A4 c.919-2A>G monoallelic mutation from 1065 hearing-impaired subjects and reported the occurrence of a second mutant allele in these patients.

**Methods:**

The occurrence of a second mutant allele in these 80 patients with a single c.919-2A>G mutation was investigated. Mutation screening was performed by bidirectional sequencing in SLC26A4 exons 2 to 6 and 9 to 21.

**Results:**

We found that 47/80 patients carried another SLC26A4 c.919-2A>G compound mutation. The five most common mutations were: p.H723R, p.T410M, 15+5G>A (c.1705+5G>A), p.L676Q and p.N392Y. We found a Chinese-specific SLC26A4 mutation spectrum and an associated SLC26A4 contribution to deafness.

**Conclusion:**

Our study illustrates that mutation analysis of other SLC26A4 exons should be undertaken in deaf patients with a single heterozygous SLC26A4 mutation. Moreover, a model of compound heterozygosity may partially explain the disease phenotype.

## Background

Hearing loss is the most prevalent form of sensory impairment in humans. Approximately 1 in 1,000 children are born with hearing loss severe enough to require special education services [1]. Deafness in at least half of all infants with profound hearing loss can be attributed to genetic factors; of these, nonsyndromic recessive causes comprise approximately 75–80% [1]. The gap junction beta-2 (GJB2) gene, which encodes connexin 26 (a component of gap junctions), is the most common cause of sensorineural hearing loss (SNHL). Mutations in the GJB2 gene are responsible for about 50% of recessively-inherited hearing loss in many populations [2].

The SLC26A4 gene, which encodes pendrin, is the second leading cause of autosomal recessive deafness characterized by congenital deafness or progressive pre- or postlingual hearing impairment [3-6]. The SLC26A4 gene is composed of 21 exons within 2343-bp cDNA on chromosome 7q22-q31 [7]. Mutations in the SLC26A4 gene are found in not only patients with Pendred syndrome (PS; OMIM # 274600), but also in individuals afflicted with nonsyndromic hearing loss (NSHL) with enlarged vestibular aqueduct (EVA) at the DFNB4 locus [5,7-9]. SLC26A4 gene studies have revealed that pendrin is a transmembrane anion transporter that is highly expressed in the inner ear and functions as an iodide/chloride transporter [10]. Pendrin is also a microtubule-associated structural protein that is likely involved in regulation of endolymphatic fluid pH through the secretion of HCO_3_^-^ ions [11]. SLC26A4 mutations associated with nonsyndromic sensorineural hearing loss with EVA showed partial transport function of pendrin, which may be sufficient to maintain thyroid function and eliminate goiter [12].

To-date, more than 170 SLC26A4 sequence variations have been identified (Pendred/BOR HomePage, http://www.healthcare.uiowa.edu/labs/pendredandbor/slcMutations.htm), including missense, frame-shift, splice-site, nonsense, and small-deletion mutations. These mutations cover all 21 exons of the SLC26A4 gene. However, the c.919-2A>G (IVS7-2A>G) mutation, which may cause deletion of exons surrounding a splice site, is a hotspot mutation in the Chinese population [13].

We established a nationwide DNA repository of samples from deaf probands to pursue a sequential screening strategy to identify gene mutations for deafness and to inform etiological diagnoses in China. This strategy yielded an estimation of the prevalence of SLC26A4 c.919-2A>G in deaf individuals in a large Chinese population [14]. Previous work revealed that the frequency of c.919-2A>G monoallelic mutations was 7.6% in our sample of the Chinese deaf population [14]. Moreover, only two other mutations (*i*.*e*., c.915insG and c.918+44delACA) were identified in addition to c.919-2A>G by direct sequencing analysis of SLC26A4 exons 7 and 8 with intron 7 in 1552 SNHL patients in China. Therefore, c.919-2A>G accounts for 99% of all mutant alleles in SLC26A4 exons 7 and 8 (278/280) [15].

The presence of two SLC26A4 mutations (*i*.*e*., homozygous or compound heterozygous; subsequently referred to as biallelic) is associated with NSHL and EVA [6,16,17]. Biallelic mutations in the SLC26A4 gene are a common cause of inner ear malformations and defective endolymph homeostasis [18,19]. Recently, two studies carried out in PS and non-PS (EVA–Mondini) patients were published. In the first [20], the authors reported that PS patients were more likely to have two mutations, as opposed to one or none in non-PS (EVA–Mondini) patients. In the second study [17], patients with biallelic mutations had more severe deafness, an earlier age of onset, and a more fluctuating course of hearing levels than patients in whom no mutation was identified.

Interestingly, 38 deaf individuals with homozygous c.919-2A>G mutations underwent temporal bone computed-tomography (CT) scans; all were confirmed to have EVA. A definite etiologic diagnosis could be made using molecular diagnostics in individuals with concomitant hearing impairment and biallelic SLC26A4 mutations. However, the incidence of c.919-2A>G compound heterozygosity among SNHL patients remains unknown. The number of cases investigated to-date is limited and the contribution of c.919-2A>G compound heterozygotes in the general deaf population is unclear. In this study, we evaluated 80 patients with the SLC26A4 c.919-2A>G monoallelic mutation from 1065 hearing-impaired subjects and reported the occurrence of a second mutant allele in these patients.

## Methods

### Subjects

This study was performed according to a protocol approved by the ethics committee of the Chinese PLA General Hospital (equivalent to the Institutional Review Board in Western nations). Informed consent was obtained from parents of students <18 years old or from adults >18 years old before blood sampling. Parents were interviewed to obtain family history and maternal pregnancy history, in addition to the subject’s age at diagnosis and clinical history, including infections, possible head or brain injuries, and exposure to aminoglycoside antibiotics.

A retrospective review of 1065 patients with moderate-to-profound bilateral sensorineural hearing loss at the Chinese PLA General Hospital Genetic Testing Center for Deafness revealed that the numbers of patients with homozygous and heterozygous c.919-2A>G were 53 (5.0%) and 80 (7.5%) by polymerase chain-reaction (PCR)/restriction-fragment-length polymorphism [14]. We evaluated the 80 patients with a c.919-2A>G single allelic mutation and found no evidence of syndromic hearing impairment. In these samples, no pathogenic mutation had been identified by sequencing in GJB2.

A total of 82 unrelated Han subjects with normal hearing sensitivity served as a control group. We used the same screening method. The controls had no family history of hearing or speech disorders.

### DNA isolation and sequencing

Mutation screening was performed by bidirectional sequencing on DNA extracted from whole blood and amplified by PCR.

### Primer design and PCR amplification

Coding and flanking intron sequences of SLC26A4 exons 2 to 6 and 9 to 21 (GenBank accession number NT_007933) were amplified by PCR using 17 primer pairs (the common pairs of exons 11 and 12) [21]. PCR reactions were performed in a 20-μL volume containing 100-ng DNA, 2 μM of each forward and reverse primer, 200 μM dNTPs, 1× PCR buffer (10 mM Tris–HCl, pH 8.3, 50 mM KCl, 2.5 mM MgCl_2_), and 1 U of DNA polymerase (Huamei, Beijing, China). PCR amplifications were performed in a Gene Amp PCR System 9700 (Applied Biosystems, Foster City, CA, USA) with an initial denaturation step at 94°C for 1 min and then 34 cycles at 94°C for 1 min, 60°C for 1 min, 72°C for 2 min, followed by 2 min of final extension at 72°C. The PCR products were purified and sequenced using the BigDye Terminator Cycle Sequencing kit (version v3.1) and ABI 3100 automated DNA sequencers (Applied Biosystems, Foster City, CA, USA) with Sequence Analysis Software (Sequencing Analysis version 3.7). DNA sequence variations were identified per reference sequences. The sequences were analyzed using the Genetool Lite software and the SLC26A4 GenBank sequence (ACCESSION: NT_007933, VERSION: NT_007933.14 GI: 51493052) ( http://www.ncbi.nlm.nih.gov/nuccore/51493052).

### Statistical analysis

Pearson’s ***x***^***2***^ test was used to evaluate the significance of allelic associations. When the theoretical frequency was <5, Fisher’s exact test was used. Pearson’s ***x***^***2***^ test was also used to compare differences in the frequencies of mutations and exons. Statistical analyses were performed using the Stata software, version 7.0 (Stata Corporation, College Station, TX, USA).

## Results

Our study sample consisted of 46 males and 34 females aged 4–20 years (mean = 12.24 ± 4.76 years). SLC26A4 mutation screening was completed in all patients with a single SLC26A4 c.919-2A>G mutation. A double-heterozygous genotype was found in 47 of 80 patients with a single c.919-2A>G mutation (58.8%), whereas 33 patients were heterozygous for c.919-2A>G without other mutations.

Mutation data on the 80 Chinese subjects are shown in Table 1. In one proband, an A to T substitution at position 426 in exon 5 was found which not leading substitution of Proline at codon 142 in the third transmembrane domain of SLC26A4. The silent mutation and the three intron variants (*i*.*e*., intron11+47T>C, intron2-14C>T and intron18-13T>A) were not considered biallelic SLC26A4 mutations.

**Table 1:** Genotype and frequencies of 47 patients with c.919-2A>G and second mutations in SLC26A4

**Mutation**	**Amino acids**	**No. of alleles (% frequency)**
c.279T>A*	p.S93R	1 (1.25%)
c.281C>T	p.T94I	1 (1.25%)
c.415+2T>C*	Splice site	1 (1.25%)
(4+2T>C)		
c.600+2T>A*	Splice site	2 (2.5%)
(5+2T>A)		
c.665G>T*	p.G222V	1 (1.25%)
c.1079C>T	p.A360V	1 (1.25%)
c.1174A>T	p.N392Y	***3 (3.75%)***
c.1226G>A	p.R409H	2(2.5%)
c.1229C>T	p.T410M	***4 (5%)***
c.1545-2 A>G*	Splice site	1 (1.25%)
(14-2A>G)		
c.1548insC	Frameshift	1 (1.25%)
c.1586T>G	p.I529S	1 (1.25%)
c.1705+5G>A	Splice site	***4 (5%)***
(15+5G>A)		
c.1825delG*	Frameshift	1 (1.25%)
c.1829C>A	p.S610X	1 (1.25%)
c.1991C>T	p.A664V	1 (1.25%)
c.2027T>A	p.L676Q	***4 (5%)***
c. 2035-1G>A*	Splice site	1 (1.25%)
(18-1G>A)		
c.2168A>G	p.H723R	***16 (20%)***

Alleles in bold italic type had frequencies >2%. *, novel mutation.

A total of 19 mutations were identified, comprising seven novel and 12 known mutations (Table 1). These mutation sites were located in the coding region that spanned 13 exons, except for exons 1, 2, 11, 12, 13, 16, 20, and 21, in which no mutations were identified. In addition to exons 7+8, which contained c.919-2A>G, three exons (exons 19, 10 and 17) and the flanking sequences of exon 15 had more mutant alleles than did others. The most common mutations, including p.H723R, had a frequency of 20% (16 of 80). The next most-common allele variants were p.T410M, 15+5G>A (c.1705+5G>A) and p.L676Q (4 of 80, 5%). The difference between p.H723R and p.T410M was statistically significant (*p* = 0.004). The fifth most-common mutation was p.N392Y (3 of 80, 3.75%). The 14 remaining mutations were detected only once or twice, with frequencies of 1.25 and 2.5%. Our data suggest that these four exons (exons 19, 10, 17 and 15) should be the priority for SLC26A4 mutation screening.

We found ten types of missense mutations, five types of splice-site mutations, two types of frameshift mutations, and one type of nonsense mutation (Table 1). We considered these SLC26A4 allele variants as potentially pathologic in causing NSHL. We tested at least 82 controls with normal hearing for all these mutations to determine whether we should exclude mutations that were common polymorphisms. None of the 19 mutations were detected in 82 normal subjects.

All the novel missense mutations occurred in the conserved regions of the coded amino acid sequences, identified by the high sequence homologies among human, mouse, and rat. Although translated protein can be made in the presence of missense mutations for some amino acid substitutions, missense mutations may result in pendrin protein dysfunction.

Nine patients were compound heterozygous for splice site mutations with c.919-2A>G. We considered these mutations to be disease-causing factors. As the five splice site mutations (*i*.*e*., 4+2T>C (c.415+2T>C), 5+2T>A (c.600+2T>A), 14–2 A>G (c. 1545–2 A>G), 15+5G>A (c.1705+5G>A)and 18–1 G>A (c. 2035-1G>A)) changed a conserved nucleotide in the splice-site consensus sequence, these mutations likely affect SLC26A4 gene splicing. The c.1825delG mutation led to a predicted frameshift at position 608 of the pendrin protein stop codon at position 634. Nonsense mutation of the c.1829C>A substitution was found in exon 17, leading to p.S610X. The frameshift mutation, c.1548insC, was identified by Park *et al*. in 2005 [16].

### Mutations found in unaffected controls

We found intron11+47T>C variation had a higher carrier frequency, with a frequency 14.6% (Homo5 and Hete7). A variation intron 19-25T>A was identified three times (Homo1 and Hete2). The c.919-2A>G heterozygous mutation was detected once in 82 normal subjects. Eight variations [*i*.*e*., c.147C>G Hete (S49R), intron 2-32A>T Hete, c.340G>A Hete (p.G114R), intron 7-18T>G Hete, c.1927G>T Hete (E643X), c.1975G>C Hete (V659L), intron 17-26A>G Hete, and c.2283A>G Homo (p.T761T)] were identified once. None of the 19 mutations was detected in 82 normal subjects. Allelic frequencies were significantly different between patients and control subjects for all five mutations (p.H723R, p.T410M, 15+5G>A (c.1705+5G>A), p.L676Q and p.N392Y; Fisher’s exact test, *p* = 0.000, 0.006 and 0.023, respectively).

## Discussion

The progress made in research on hereditary hearing loss indicates the importance of molecular diagnosis using the SLC26A4 gene in patients with hearing loss [22]. The molecular diagnosis of hearing impairment associated with the SLC26A4 gene is reliable because deafness is caused by homozygous or compound heterozygous SLC26A4 mutations [6,23]. Previous studies [13,24,25]. suggested that SLC26A4 c.919-2A>G is the most frequent mutation in Taiwan and mainland China, and that less-frequent mutations of other exons may be detected by direct DNA sequence analysis [26]. In the present study, 58.8% of patients carrying a heterozygous c.919-2A>G mutation (47/80) had a definite form of SNHL caused by a SLC26A4 mutation. We also found that compound heterozygosity accounted for nearly 60% of patients with two mutated alleles, which highlights the importance of screening for SLC26A4 mutations other than c.919-2A>G.

In this study, 19 mutations were identified, seven of which were novel. Of these mutations, 16 were found only in the Asiatic deaf population, whereas three were identified in both Asians and Caucasians. It seems that the spectrum of SLC26A4 mutations in the Chinese population is different from that of European populations. The most common allele variant was p.H723R, situated within exon 19, which accounted for 34% of all mutant alleles. P.H723R appeared to be highly correlated with nonsyndromic recessive deafness with EVA among Chinese populations [13]. Park *et al*. [27] reported that the p.H273R mutation is prevalent; this allele exists in the majority of SLC26A4 mutations in Korean and Japanese populations. The second-most-frequent mutations (*i*.*e*., p.T410M, 15+5G>A (c.1705+5G>A), and p.L676Q) occur at exon 10, exon 15 flanking sequences, and exon 17, respectively. Exons 19, 10, 17 and 15 (flanking sequences) have highly variable region(s) in which multiple mutations were detected. Therefore, this study showed that mutations other than c.919-2A>G exist mostly in four exons (*i*.*e*., exons 19, 10, 17 and 15). The mutation frequency in SLC26A4 exons 19, 17, 10, and 15 was consistent with the results of previous studies [13]. However, our study was performed on patients with severe to profound hearing impairment or deafness (general deaf population), whereas Wang investigated patients with hearing impairment and EVA/MD [13]. The distribution of mutant SLC26A4 alleles revealed by our study suggests that mutation screening of four exons (*i*.*e*., exons 19, 10, 17 and 15) following c.919 A>G identification should be the priority for NSHL genetic testing, especially in EVA/MD patients.

We found seven SLC26A4 mutations that have not been described previously. These included two missense mutations, four splice-site mutations, and one frameshift mutation. The missense mutations were probably pathogenic, based on both their presence in affected individuals and their predicted biological consequences. That is, a change in an amino acid that is located in a functional domain or that is conserved in related genes or species is likely to be pathogenic. The missense mutations p.S93R and p.G222V are most likely to be pathogenic based on the changes in evolutionarily conserved amino acids, Serine to Arginine and Glycine to Valine, respectively. However, as the SLC26A4 gene is expressed only in thyroid, kidney, and brain [7,12,28], and since none of these tissues was available from patients, we were unable to evaluate the effect of the mutation at the mRNA level. The splice site mutation is frequently found in compound heterozygotes with a c.919-2A>G mutation [29]. The pathogenic potential of splice site mutations is unknown since their effect on splicing has not been determined. We presume that these splice site mutations could cause exon deletion and affect SLC26A4 mRNA integrity and pendrin function. The SLC26A4 frameshift mutation (c.1825 delG) was a nonsense mutation, resulting in an unstable mRNA or truncated protein.

A second mutant allele was not detected among 33 patients in our study. It is unlikely that a single SLC26A4 mutant allele is sufficient to cause EVA, as there are no published reports of vertical co-segregation of EVA with a single SLC26A4 mutant allele or sporadic cases associated with a single *de novo* SLC26A4 mutant allele [30]. In a previous study, we performed single mutation screening of deaf individuals from Chifeng and Nantong Cities, and found one mutant allele mutation in SLC26A4 was identified in 8.45%(24/284) [31].

It still remains unclear whether or why the heterozygous c.919-2A>G mutation affects the inner ear. It can be assumed that patients with a single c.919-2A>G mutation are more likely to develop hearing loss and EVA in the presence of additional genes or environmental factors. On the basis of current knowledge, we speculate that other regulatory factors or genes are involved in the production or activity of the inner ear proton pump or that environmental factors affect pump function in some patients. First, a subset of individuals with a monoallelic SLC26A4 gene mutation might harbor a second unidentified mutation in the promoter or intronic regions of this gene. It has been demonstrated that Foxil-null mice have both enlarged endolymphatic ducts/sacs and hearing loss, and Foxil is a possible upstream regulator of SLC26A4 [32]. Yang *et al*. described several EVA patients with a heterozygous SLC26A4 mutation in combination with a heterozygous hypo-functional variant of FOXI1 or KCNJ10 [33,34]. Mutations in introns that may activate cryptic splice sites might be present in human populations. Second, since deafness is associated with GJB2 and GJB3 [35], it should be noted that the deafness associated with the SLC26A4 gene might be a consequence of digenic inheritance. Last, environmental or unknown factors may explain the phenotype of EVA patients with a single heterozygous c.919-2A>G mutation. Alternatively, SLC26A4 epigenetic modifications, such as DNA methylation, might repress gene transcription and account for the observed co-segregation of deafness and SLC26A4 monoallelic mutations [36].

We propose a new algorithm for genetic screening in association with SLC26A4 mutations, based on the frequency of mutations in different exons. In SNHL patients, the SLC26A4 gene should be examined by preferential exon screening. If results are inconclusive, then we propose mutation screening in sequence, since the four most frequent types of mutations account for the majority mutant alleles (66%) in our experience (Table 1). If no mutations are detected in exons 19, 10, 17 or 15, then analysis of other SLC26A4 exons should be undertaken. This algorithm should detect almost two thirds of mutant alleles and is particularly useful for genetic screening in hearing loss patients.

EVA is associated with characteristic clinical findings, including fluctuating and sometimes progressive sensorineural hearing loss. Hearing loss in some EVA patients is mild to moderate. This study was limited by the moderate-to-profound hearing loss requirement, which decreased EVA patient enrollment. However, our findings show that biallelic SLC26A4 mutations are a common cause of moderate-to-profound hearing loss. Our results underscore the importance of a genetic screening program that is capable of detecting different SLC26A4 mutations. The screening of SLC26A4 gene mutations will assist us with genetic counseling, and screening for SLC26A4 gene mutations in patients with moderate or profound hearing loss should be routine. Furthermore, our data provide information that will foster genetic approaches to early diagnosis in the Chinese population.

## Conclusion

In summary, 58.8% of the SLC26A4 IVS7-2A>G mutation patients examined had clear SLC26A4-induced SNHL. Our evaluation of SLC26A4 mutations provides a basis for improving the clinical diagnosis of deaf patients that may also in future be used in prenatal genetic analysis. Because CT or magnetic-resonance imaging (MRI) is often difficult to perform in newborns, genetic testing allows clear diagnosis and effective early treatment, such as hearing aids or cochlear implantation. SLC26A4 mutation screening can dramatically improve the identification of causality, associated genetic counseling, and, to some extent, patient management. Moreover, this observation highlights some of the difficulties in making a definitive genetic diagnosis for deafness and illustrates some of the challenges that may be associated with molecular genetic testing. The causes of EVA in patients with nondiagnostic SLC26A4 genotypes are largely unknown. Identification of additional genes or factors underlying EVA would facilitate a more precise diagnosis and could provide mechanistic insight into the pathogenesis of hearing loss in this enigmatic disorder.

## Competing interests

The authors declare that they have no competing interests.

## Authors’ contributions

QL carried out the molecular genetic studies, participated in the sequence alignment and drafted the manuscript. QZ, YY and SH participated in the sequence alignment. DYH and DLH participated in the design of the study and performed the statistical analysis. PD conceived of the study, and participated in its design and coordination and helped to draft the manuscript. All authors read and approved the final manuscript.
